# Community Science: Big Insights From Small Mammal Data

**DOI:** 10.1002/ece3.73506

**Published:** 2026-04-17

**Authors:** Marion Sherbourne, Meghan Ward, Meagan Stager, Shelby Cohen, Lucy van Haaften, Thomas Burgess, Bryan Hughes, Jeff Bowman

**Affiliations:** ^1^ Environmental and Life Sciences Graduate Program Trent University Peterborough Ontario Canada; ^2^ Biology Graduate Program Acadia University Wolfville Nova Scotia Canada; ^3^ Wildlife Research and Monitoring Section Ontario Ministry of Natural Resources and Forestry Peterborough Ontario Canada

**Keywords:** abundant, citizen science, cryptic, enigmatic, iNaturalist, rare

## Abstract

Biodiversity surveys can be costly and labor‐intensive; therefore, community science databases serve as a practical resource to support survey goals. Community science datasets may be a valuable tool for easily identifiable species. In contrast, cryptic or hard‐to‐identify species may appear less frequently in community science datasets. We investigated the reliability of community science datasets for evaluating the distribution and abundance of small mammals in Ontario, Canada. We selected eight focal species, categorized by historical abundance and ease of identification. We ran a community outreach event from May to August 2025 to promote the contribution of species records as a tool for scientific research. We collected data directly from iNaturalist, the Global Biodiversity Information Facility (GBIF), and records provided directly to us by community members. We counted observations of each focal species before and after our outreach campaign to assess the effectiveness of community outreach for small mammals. Our campaign did not increase the number of iNaturalist submissions of our focal species compared to previous time windows of a similar duration. Furthermore, few observations were obtained for rare or confusible species, whereas most were of abundant, non‐confusible species. The lack of abundant new iNaturalist records in response to our campaign may reflect a broader gap in photographic evidence to support the identification of rare small‐mammal species. Despite the low number of new iNaturalist records, the combined datasets dramatically increased the total number of records. Our study highlights the limitations of using community science repositories to monitor rare and confusible species. Integrating other community sources alongside iNaturalist yielded the most robust dataset. We encourage small‐mammal researchers and enthusiasts to submit photographs of rare or confusing species to community science repositories and to validate submitted photographs. We also suggest establishing a verification process to include community science records without pictures.

## Graphical abstract

Community science databases can support biodiversity surveys, especially for easily identifiable species, but they are less effective for rare or cryptic species. In a study of small mammals in Ontario, Canada, a community outreach campaign did not significantly increase iNaturalist submissions, and most records were of common, easily identifiable species. However, combining multiple community datasets greatly expanded the total data, highlighting the need for verified submissions and additional sources to better monitor rare or confusable species. Photo credit goes to Jacob Bowman.

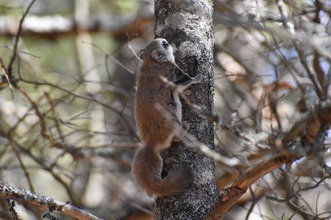

## 1 Introduction

Globally, climate change, habitat destruction, overexploitation of resources, and ongoing land‐use changes have exacerbated biodiversity decline (Ray et al. 2021; Rull 2021). Monitoring biodiversity is essential for understanding ecological processes, detecting population trends, and guiding conservation action (Stephenson et al. 2022). Across taxa, both short‐ and long‐term monitoring have revealed patterns of diminishing biodiversity, range expansion, and shifting distributions in response to changing environmental factors (Bowman et al. 2005; Turley et al. 2022). Monitoring programs are often limitedowever, in both taxonomic and spatial coverage (Boakes et al. 2010; Proença et al. 2017). Thus, researchers and policy makers benefit from the inclusion of non‐structured data, including community science (Think Box 1) initiatives, that can be used to assess a broader spectrum of taxa and geographic locations (Chandler et al. 2017). Community‐science datasets help to provide the foundation for conservation policy and management, yet they are unevenly spread across taxa (Mair and Ruete 2016). Taxa that are large, and non‐confusible (Think Box 1), or charismatic species, often benefit the most from extensive biodiversity assessment efforts (Tensen and Teske 2025). In contrast, smaller, and more confusible species, or taxa that are rare, remain poorly studied, and are therefore underrepresented in wildlife management efforts (Droghini et al. 2022; Troudet et al. 2017).

THINK BOX 1 **Definitions.**

1. Community science: the contribution to, and/or production of scientific research, monitoring, and/or knowledge by any members of a community, without regard to citizenship or borders (adapted from Houllier and Merilhou‐Goudard 2016 and Charles et al. 2020).

2. Confusible: species that lack distinct morphological traits or are difficult to notice, which hinders accurate identification by most community members.

3. Non‐confusible: a species easily identified at a glance, with distinct morphological traits to aid in accurate identification by most community members.

Species that are more difficult to identify or are observed less frequently are still important for conservation efforts. For example, small rodents that are generally confusible and usually absent during daylight when most non‐research wildlife observations occur are important components of the ecosystem. Small rodents are seed dispersers, disease vectors, and are important prey items for many carnivores (Mukherjee et al. 2004; Morand et al. 2015; Brehm and Mortelliti 2022). Many rare or confusible small mammal species lack robust population monitoring due to their low detectability and the frequent challenges of species‐level identification (Jetz and Freckleton 2015; Peralta et al. 2022; Boonstra et al. 2025). A lack of data can lead to a lack of active conservation efforts and policy guidelines for these species (Parsons 2016; Sebsibe Tafesse and Berihun Yohannes 2022; Medd et al. 2025). For example, under the Canadian Species‐at‐Risk Act, species assessments are deferred for species considered data deficient (COSEWIC 2021).

### 1.1 Community Science as a Tool

Community science can contribute to biodiversity monitoring, as local events focused on education and community involvement, or open repositories such as eBird and iNaturalist encourage broad participation. These biodiversity datasets can be produced at temporal and spatial scales that traditional ecological studies cannot easily achieve due to factors such as limits to property access or funding (Bonney et al. 2009; Brown and Williams 2018). Community science programs increase the reach of ecological monitoring, facilitate rapid responses to conservation issues, and encourage participation in science by engaging volunteers in documenting species observations. Platforms such as eBird and iNaturalist now contain millions of location‐specific records, which have been applied to questions ranging from species distribution modeling and biodiversity assessments to tracking the spread of invasive species and rediscovering taxa once thought extinct (Jones et al. 2019; Wilson et al. 2020; Baici and Bowman 2023; Mason et al. 2025). These contributions are especially valuable in the context of global biodiversity loss, where broad participation can generate critical data on threatened or poorly documented species that are otherwise difficult to monitor (Fontaine et al. 2022).

Community science has demonstrated unique strengths in documenting both rare and confusible species (Mesaglio et al. 2021; Roberts et al. 2022). Because rare taxa are often sparsely distributed and overlooked by conventional surveys, opportunistic reports from volunteers can provide critical insights into their status (Zeng et al. 2018; Báthori et al. 2022). For example, Báthori et al. (2022) showed that a supposedly rare ant species ( *Cryptopone ochracea* ) was readily detected through online naturalist groups, leading to new distribution records across Hungary and Serbia. Similarly, Zeng et al. (2018) coordinated a community science survey that clarified the wintering range of the endangered scaly‐sided merganser ( *Mergus squamatus* ) in China. Social media‐based efforts have also produced unexpected records of rare and easily recognizable species such as the ornate eagle ray ( *Aetomylaeus vespertilio* ), where distinctive markings facilitated reliable identification from opportunistic photographs (Araujo et al. 2020). These cases illustrate how community science and opportunistic observations can compensate for the challenges posed by rarity or elusiveness by greatly increasing the scale of the search effort.

### 1.2 iNaturalist as a Case Study

iNaturalist is among the most widely used community science platforms, with contributions from millions of observers worldwide (Seltzer 2019). Observations are supported by photo or audio evidence, artificial intelligence‐assisted identifications, and a community review system that can yield research‐grade records suitable for scientific use where there is a photographic or audio record (Campbell et al. 2023). The platform has contributed to updating range maps (Jensen et al. 2025), species rediscoveries (Jones et al. 2019), and improved understanding of species interactions (Gazdic and Groom 2019). Despite the broad uptake of iNaturalist across taxa, there have been limited assessments of its effectiveness for cryptic fauna, such as many small‐mammal species (Kays et al. 2022; Alyetama et al. 2025; Herrera et al. 2025).

### 1.3 The Challenge of Small Mammals

Small mammals are a diverse assemblage that includes rodents, shrews, and other species weighing under 5 kg (Merritt 2010). Despite their diversity, many are difficult to monitor because they are morphologically similar, elusive in behavior, or rarely encountered (Kays et al. 2022). For example, voles, shrews, and mice can often be broadly distinguished from each other using photographs; however, species‐level identification from opportunistic records is often more uncertain due to poor photograph quality and strong morphological similarities (Kays et al. 2022). Species‐level identification for many small‐mammal taxa requires expert‐led training (Brown and Williams 2018). Subtle differences in morphology used for identification suggest that not all small mammals are equally suited to community science approaches, as even expert‐trained individuals can have trouble identifying small mammals through photographs or ephemeral sightings.

Previous work demonstrates both opportunities and limitations of community scientists in small‐mammal research and monitoring. Torre et al. (2021) reviewed programs in Catalonia that relied on standardized live‐trapping protocols carried out by volunteers, which provided valuable insights into distribution and abundance, but required significant program structure and did not use iNaturalist. Similarly, Parsons et al. (2018) showed that trained volunteers could successfully deploy and interpret camera traps for mammal surveys: an initiative that again differed from the opportunistic and unstructured contributions typical of iNaturalist submissions. These studies show that community science can generate robust data on small mammals, but also highlight challenges associated with rare or confusible species, and the existing research gap in knowledge about the suitability of platforms like iNaturalist for this role.

The effectiveness of iNaturalist for collecting small mammal data is likely to depend on both abundance and confusibility. Abundant and non‐confusible species may be overrepresented because they are common and distinctive (Callaghan et al. 2021; Tensen and Teske 2025). In contrast, we suspect that rare and confusible species are the least likely to be represented in iNaturalist data, as they are seldom encountered and difficult to identify from opportunistic field photographs (Ozolina et al. 2025). When integrated with careful validation and contextual information, community science offers a cost‐effective and powerful means of improving data for species that are otherwise difficult to study (Brown and Williams 2018; Pernat et al. 2024). Rare and confusible species may be a gap, however, in community science efforts, since species may be misidentified or missed altogether (McMullin and Allen 2022). Considering these potential gaps is essential for evaluating whether iNaturalist and similar approaches can provide meaningful insight into small mammal abundance and distribution.

### 1.4 Rationale and Study Objectives

The potential of community science for rare and confusible species has been demonstrated in other taxonomic groups including the scaly‐sided merganser ( *Mergus squamatus* ; Báthori et al. 2022) and ants (*Crptopone orhracea*; Zeng et al. 2018). However, there is limited consensus on how effectively platforms like iNaturalist can be applied to evaluate the distribution and abundance of small mammals. Addressing this gap is important for both practical and conceptual reasons: small mammals are frequently overlooked in conservation planning, and biases in opportunistic data collection may obscure patterns of rarity, abundance, and distribution. By explicitly considering whether species are rare versus abundant and confusible versus non‐confusible, we can test how these species traits shape representation in iNaturalist datasets.

We evaluated the application of iNaturalist and community datasets (such as the Global Biodiversity Information Facility (GBIF)) for surveying small mammals, focusing on both confusible and non‐confusible species, as well as rare and abundant species. Community science platforms offer the potential to generate large‐scale ecological data, but their effectiveness may vary depending on species detectability and population size. Understanding these limitations is critical for interpreting observations and for designing targeted monitoring campaigns. We aimed to determine whether community science platforms can provide reliable data for confusible species and whether targeted campaigns increase the number of research‐quality observations. We also assessed the value of targeted community outreach and the inclusion of community datasets in addition to iNaturalist records for improving data for our species of interest.

We predicted that confusible species would be underrepresented relative to more non‐confusible species, reflecting the challenges of observation. Additionally, we predicted that there would be a positive association between the number of observations and species abundance, a positive association between the number of observations and non‐confusibility, and that the magnitude of effect would vary depending on the combination of these features (Table 1). This framework allowed us to evaluate how well research‐grade iNaturalist observations capture abundance patterns across species with varying detectability and rarity. Further, we predicted that the inclusion of additional community datasets acquired through targeted outreach and GBIF records would improve the resolution of our data.

**TABLE 1:** Predicted proportional increase in species observations based on rarity and confusibility levels.

	Rare	Abundant
Confusible	Smallest	Large
Non‐confusible	Small	Largest

## 2 Methods

### 2.1 Focal Species

For this study, we chose eight small mammal species found in Ontario, Canada, that vary in rarity and confusibility (Table 2 and Figure S3). The species were chosen to represent different combinations of rarity and confusibility, using expert opinion (Kays et al. 2022) and Ontario Natural Heritage Information Centre (NHIC) rarity rankings (Ministry of Natural Resources 2025) (Table 2).

**TABLE 2:** Classification of each focal species as either confusible or non‐confusible, and rare or abundant. For each species of interest, distinguishability from other sympatric species (confusible vs. non‐confusible) is based on expert opinion (Figure S1). NHIC ranks (Ministry of Natural Resources 2025) range from SU: Species is unrankable due to lack of information, SH: Species is possibly extirpated, and S1: Species is critically imperiled to S5: Species is secure.

Common name	Latin name	NHIC rarity rank	Rarity classification	Confusibility classification
Star‐nosed mole	*Condylura cristata*	S5	Abundant	Non‐confusible
Northern short‐tailed shrew	*Blarina brevicauda*	S5	Abundant	Non‐confusible
Northern flying squirrel	*Glaucomys sabrinus*	S5	Abundant	Confusible
American water shrew complex	*Sorex palustris*	S5	Abundant	Confusible
Least weasel	*Mustela nivalis*	SU	Rare	Non‐confusible
Eastern mole	*Scalopus aquaticus*	S2	Rare	Non‐confusible
Least shrew	*Cryptotis parva*	SH	Rare	Confusible
Woodland vole	*Microtus pinetorum*	S3	Rare	Confusible

### 2.2 Outreach

To encourage submissions for targeted species, we conducted an Ontario‐wide outreach campaign aimed at community member‐led naturalist groups, provincial and national parks, and various environmental organizations across Ontario. This included emailing an informative flyer to approximately 215 organizations across the province during spring 2025, prior to the start of the data collection window (see Figure S1 for flyer and Figure S2 for email text). Our correspondence indicated that we would collect submissions from May 1, 2025, to August 31, 2025. Small mammals are generally more active in warmer temperatures which increases their detectability, and most observations tend to be posted to iNaturalist in the summer months (iNaturalist 2025), making May to August an ideal time for our campaign. We invited participants to contribute both historical and recent observations of small mammals to iNaturalist, emphasizing that all records, including single observations from under‐reported areas, were valuable. This campaign was designed to leverage the expertise, field experience, and observational skills of Ontario's naturalist community to enhance the quantity and quality of community‐generated data for the eight focal species in our study.

### 2.3 Data Sources

We obtained observation data for our focal species in Ontario from GBIF, iNaturalist, and data generously provided to us by community members. Data were downloaded on August 31, 2025. Only research‐grade observations or those submitted directly to us by field experts in response to the campaign were retained for analysis to ensure data reliability. iNaturalist observations achieve research grade status when they include a date, geographic coordinates, and a photo or audio recording, are identified to at least the species level, and have had at least two out of three identifiers agree on the same taxonomic identification (iNaturalist 2025). For the purpose of this study, we define field experts as individuals with demonstrated taxonomic or ecological expertise in the relevant taxa, such as researchers, naturalists, or experienced practitioners. Some research‐grade iNaturalist submissions are automatically included in GBIF downloads; we manually filtered the dataset to remove any duplicates.

To evaluate the effects of our targeted outreach campaign, we compared the total number of observations submitted to iNaturalist before and after May 1, 2025, to August 31, 2025, coinciding with our outreach program. As a point of comparison and to evaluate the effects of the campaign, we also calculated the average increase in submissions for each focal species from May to August during 2019–2024.

### 2.4 Data Analysis

We first conducted descriptive analyses to summarize changes in iNaturalist observations for the eight focal species before and after the outreach campaign. For each species, we calculated the total number of research‐grade observations submitted before and after the outreach campaign. We then computed the absolute and relative (percent) change in submissions over this period to quantify the effect of the targeted community science campaign. Additionally, we compared the number of observations of our focal species submitted to iNaturalist from May–August 2025, with the number of Ontario observations submitted for the same species during the same months during 2019–2024 to assess the effects of our outreach. Change in the number of observations for each focal species was calculated using the following formula:

$$\left[\left(\#\text{observations after}-\#\text{observations before}\right)÷\left(\#\text{observations after}+\#\text{observations before}\right)\right]\times 100$$

We assessed observer expertise and discounted unverifiable observations submitted by non‐experts. To calculate identification error, we counted incorrect identifications and unidentifiable observations (based on our assessment) for all observations up to the first five pages of iNaturalist records (*n* = 480) of each species. We used the proportion of incorrect or unidentifiable observations from among the manually evaluated observations, performed by the authors, to determine the rate of observation error for each species (Table 3).

**TABLE 3:** Change in research grade observations of focal species submitted to iNaturalist before and after an outreach event (May 1, 2025—August 31, 2025) and during the same period in previous years (2019–2024).

Species	Absolute # of iNaturalist observations before campaign	Absolute # of iNaturalist observations after campaign	Proportional change before/after (expressed as a %)	Average % Increase/year from May–August 2019–2024 (95% CI)	Species identification error (%; up to 480 observations)
*Abundant–Non Confusible*					
Star‐nosed mole	634	719	6.3%	11.2% (5.5, 17.2)	0.2%
Northern short‐tailed shrew	2008	2192	4.4%	8.9% (4.1, 14.1)	0%
*Abundant–Confusible*					
Northern flying squirrel	389	403	1.8%	7.5% (1.2, 14.1)	19.9%^a^
American water shrew complex	18	20	5.3%	4.4% (−1.8, 10.5)	0%
*Rare–Non Confusible*					
Least weasel	3	3	0%	16.7%^b^	0%
Eastern mole	45	49	4.3%	7.7% (−5.4, 21.2)	0%
*Rare–Confusible*					
Least shrew	0	0	0%	0%^b^	0%
Woodland vole	3	3	0%	0%^b^	0%

^a^ An additional 22.8% of research grade observations of northern flying squirrels were deemed unidentifiable by our team of experts.

^b^ Confidence Intervals could not be calculated for the least weasel, woodland vole, and least shrew due to a lack of sufficient new observations submitted from May–August of 2019–2024.

## 3 Results

### 3.1 iNaturalist Data

The proportional change in the number of focal species observations submitted in Ontario from May 1st to August 31st during 2025 was similar to that observed during the same months from the years of 2019–2024 (Table 3 and Figure S4). No new observations of rare and confusible species were generated during community outreach. Four new observations were submitted for the eastern mole (rare, non‐confusible), but no new observations were submitted for the least weasel in the same category. There were 16 new observations recorded for abundant and confusible species (American watershrew complex *n* = 2; northern flying squirrel *n* = 14), and 269 new observations were submitted for abundant and non‐confusible species (northern short‐tailed shrew *n* = 184; star‐nosed mole *n* = 85). Proportional increases varied by species and category (Table 3 and Figure S4). Identification error among research‐grade iNaturalist observations was not detected for any species, except for the star‐nosed mole (0.2%), and the northern flying squirrel (19.9%).

### 3.2 Combined Dataset

**FIGURE 1:**
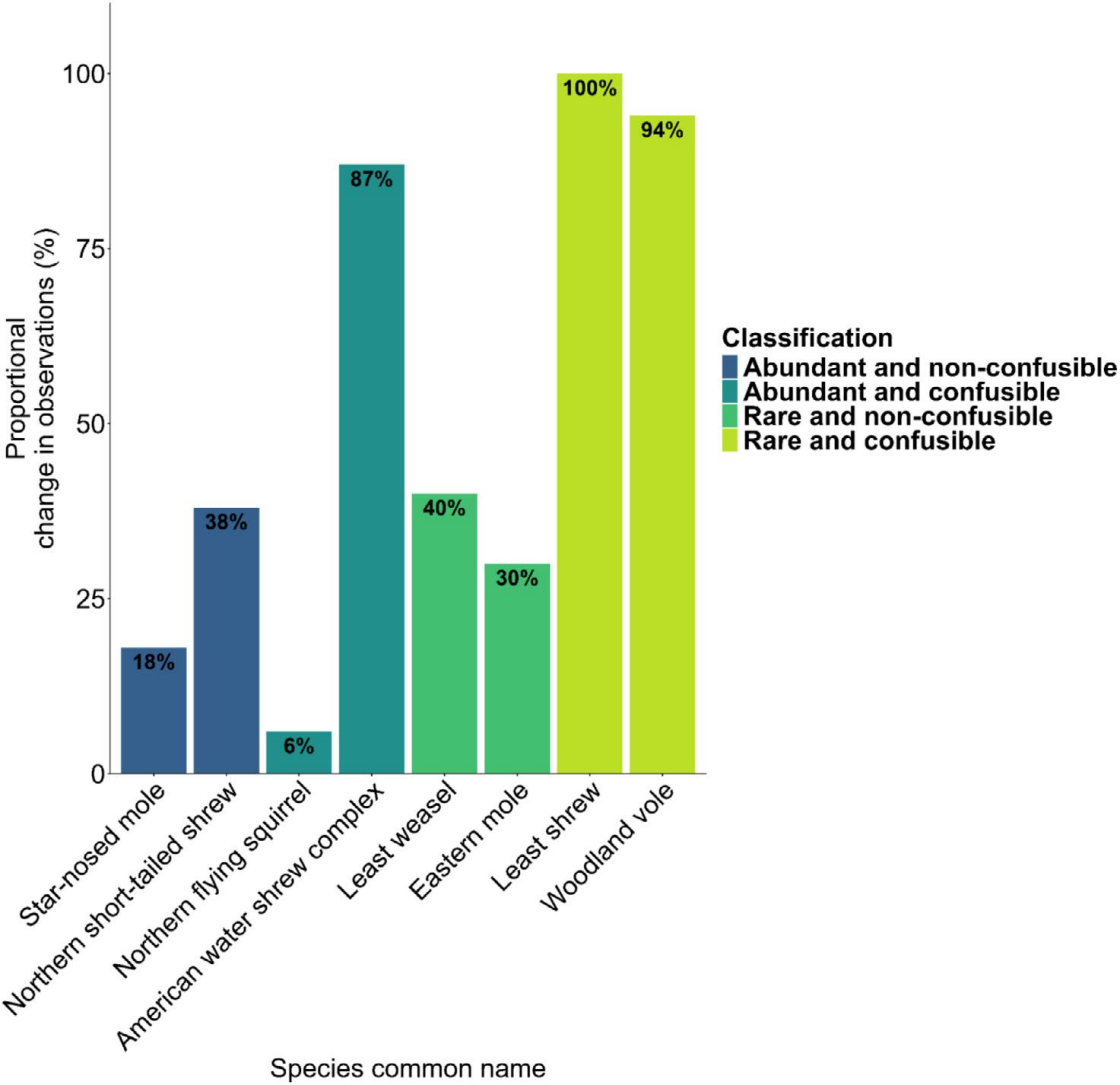
Plot of the proportional change in observations (%) comparing the number of iNaturalist observations for each focal species before community outreach (01 May 2025) to the combined community dataset after outreach (31 August 2025). This figure shows a > 90% increase in proportional change in observations in rare and confusable taxa after the outreach event, when all community data is combined.

In addition to assessing iNaturalist submissions, we also received submissions from community members via email, and we accessed the entirety of the open‐source GBIF database. We termed all of this additional information (including iNaturalist submissions, email submissions, and the GBIF dataset) as ‘combined community data’. Including this full set of combined community data increased our sample size from 3100 iNaturalist observations to 6600 total observations of our species of interest across Ontario after the outreach event (Table 4 and Figure 1).

**TABLE 4:** Comparison of iNaturalist data prior to May 01, 2025, relative to the whole community dataset (including GBIF, community records, Ontario's Ministry of Natural Resources' small mammal data) received during community outreach from 01 May 2025–31 August 2025.

Species	# of iNaturalist observations before campaign	Absolute # of whole community observations after campaign	Proportional change before/after (expressed as a %)
*Abundant—Non Confusible*			
Star‐nosed mole	634	927	18.8%
Northern short‐tailed shrew	2008	4694	40.1%
*Abundant–Confusible*			
Northern flying squirrel	389	524	14.8%
American water shrew complex	18	262	87.1%
*Rare–Non Confusible*			
Least weasel	3	7	40.0%
Eastern mole	45	84	30.2%
*Rare–Confusible*			
Least shrew	0	6	100.0%
Woodland vole	3	96	93.9%

Overall, our targeted iNaturalist community outreach from May to August 2025 did not yield any new research‐grade observations of species that were classified as both rare and confusible (Table 3). However, the combined community dataset yielded new observations for both of our rare and confusible focal species, and for both species that were rare and non‐confusible. Combined community data also yielded additional new observations of all non‐rare species (Table 4 and Figure 1).

## 4 Discussion

Our analysis supports our predictions, suggesting that the utility of iNaturalist and other community‐science platforms for surveying small mammals is strongly influenced by the rarity and confusibility of the species. Confusible and rare species were poorly represented in our community data, while non‐confusible and abundant species were far more frequently observed. No new records were generated of rare, confusible species; rare, non‐confusible species were also poorly represented, but new records were generated; abundant, confusible species demonstrated a moderate amount of change; abundant, non‐confusible species demonstrated the greatest amount of change (Table 3). The median time for research‐grade categorization on iNaturalist is around 4 h (Campbell et al. 2023). Therefore, we are confident that those submissions with potential to be research grade were verified within the four‐month window of our outreach campaign.

### 4.1 Error Rates

The negligible error rate among our non‐confusible species reinforces established claims in the literature that community science data is most reliable for easily identifiable species (Koo et al. 2022). The highest error was observed for northern flying squirrels (~20%), which is unsurprising given their high abundance and high confusibility with the southern flying squirrel ( *G. volans* ), particularly when relying on camera trap photos or those taken at a distance (McAndrew et al. 2025). High error rates for confusible species raise additional concerns about the reliability of research‐grade iNaturalist data. Although the platform's community identification system increases accuracy (Campbell et al. 2023), morphological similarity among small mammals means that even identifications corroborated by several people may be incorrect if key distinguishing traits are absent from photographs (Kays et al. 2022). The risk of misidentification increases for confusible species living in shared or overlapping habitats (Hending 2025; Kays et al. 2022).

In contrast, iNaturalist observations of the highly confusible American water shrew complex were almost entirely verified by career scientists and had no identification error. The absence of identification error among rare species is likely to result from reliance on expert identification. High error among abundant, confusible species is important to consider when relying on untrained identification and highlights the importance of accessible community identification resources and education (Brown and Williams 2018).

### 4.2 Impacts of Community Outreach

Our results clearly demonstrate the value of integrating iNaturalist records with other data sources. By combining iNaturalist observations with records from public databases such as GBIF and additional contributions provided by community members, we were able to dramatically expand the size of our dataset. This expansion improved the representation of rare species that were poorly documented in iNaturalist alone (Table 4). In 2022, Bathori et al. used community science platforms (including taxa‐based interest groups on Facebook) to gain new insight into the distribution of a rare ant species ( *Cryptopone ochracea* ). Our work complements that of Báthori et al. (2022) by highlighting the importance of integrating multiple community science platforms in community‐based research. For example, at the time of our outreach campaign, there were no research‐grade observations of the least shrew in Ontario, Canada on iNaturalist. However, when we expanded our search to include other community datasets, we found five records for the least shrew. Following the same trend, a total of three research‐grade observations for the woodland vole were found on iNaturalist; however, a total of 93 additional observations were present in the entire community‐wide dataset. Community science datasets are, therefore, most robust when multiple platforms are considered.

### 4.3 Implications for Small Mammal Surveying

Our results have important implications for the use of iNaturalist and other open‐source platforms in small mammal research. We have shown that community data can provide valuable information that supplements traditional monitoring programs for abundant and non‐confusible species; a finding that is common in the literature (Anton et al. 2018; Koo et al. 2022; Roberts et al. 2022). Our results suggest that such applications are most robust for taxa with low misidentification risk. Notably, Anton et al. (2018) found that camera trap data were accurately identified by community scientists when the mammalian fauna were already familiar to the community. Anton et al.'s (2018) results are consistent with our data, as we also found that the error rate was low when non‐confusible mammals were observed. In contrast, poor representation and higher error rates for confusible and rare species highlight the limitations of opportunistic, photograph‐based observation for monitoring small mammal groups.

Our results are corroborated by previous studies that have reported on the difficulty of applying unstructured community science data to rare or threatened species, where under‐sampling and uncertainty can hamper conservation assessments and planning (Fontaine et al. 2022; Martin et al. 2022). The bias that we observed towards abundant, non‐confusible species likely reflects that more abundant species are more likely to be encountered by opportunistic sampling, and distinctive markings facilitate more accurate, species‐level identification (Araujo et al. 2020; Koo et al. 2022). It is possible, and perhaps likely, that the lack of observations for rare species reflects actual population trends (Parsons 2016; Martin et al. 2022).

### 4.4 Future Directions

Despite some of the challenges we have identified in this study, our results reinforce the importance of including community data in species monitoring. Moving forward, we suggest the integration of iNaturalist with community‐wide data sources as tools for small mammal surveying and research, with more traditional and complementary monitoring methods like camera traps and live or hair trapping. Future research could also consider integrating additional community datasets, including owl pellet dissections from elementary and high schools (van Strien et al. 2015; Pelosi et al. 2025; Brustenga et al. 2024). Our findings align with those of Martin et al. (2022), where increasing collaboration and engagement among researchers effectively improves data collection.

While assessing the accuracy of identifications across our eight focal species, we often found that small mammal experts on iNaturalist would document an observation without including photographs. iNaturalist requires submissions to have photographic evidence to qualify as research‐grade. We therefore found that expert observations sometimes lacked research‐grade status despite their likely reliability. We recommend that all experts submit photographs with their observations to support the accumulation of research‐grade observations. Furthermore, we recommend the development of a taxon‐specific ‘expert’ profile status on iNaturalist (e.g., a blue check verified status) to facilitate observations made by experts being reported as research‐grade.

### 4.5 Conclusion

Our study reinforces that iNaturalist is a powerful community science platform for surveying non‐confusible and abundant small mammals, but its capacity to capture rare and confusible species remains limited. Our results underscore the need to interpret iNaturalist data cautiously when applying it to small mammal research. Rather than serving as a replacement for traditional methods, iNaturalist should be seen as a complementary tool that can be used to document widespread and distinctive species, while offering limited insights into rare or confusible species. Integrating community‐sourced data, including GBIF records and records provided by community members, with traditional monitoring methods may provide additional information relevant to ongoing species assessments. Future efforts should explicitly account for species rarity and confusibility when designing studies, and should explore the integration of opportunistic community science and targeted community outreach with structured monitoring. By doing so, researchers can maximize the value of iNaturalist while mitigating its limitations, contributing to more comprehensive biodiversity assessments for underrepresented taxa.

## Author Contributions

**Marion Sherbourne:** conceptualization (lead), data curation (supporting), formal analysis (lead), investigation (equal), project administration (lead), visualization (equal), writing – original draft (equal), writing – review and editing (lead). **Meghan Ward:** conceptualization (lead), funding acquisition (supporting), investigation (lead), project administration (lead), writing – original draft (lead), writing – review and editing (supporting). **Meagan Stager:** conceptualization (equal), formal analysis (equal), methodology (equal), project administration (equal), visualization (equal), writing – original draft (equal), writing – review and editing (equal). **Shelby Cohen:** conceptualization (equal), data curation (equal), formal analysis (equal), project administration (equal), visualization (equal), writing – original draft (equal), writing – review and editing (equal). **Lucy van Haaften:** conceptualization (equal), data curation (equal), formal analysis (equal), project administration (equal), visualization (equal), writing – original draft (lead), writing – review and editing (equal). **Thomas Burgess:** conceptualization (equal), data curation (lead), formal analysis (equal), project administration (equal), visualization (supporting), writing – original draft (supporting), writing – review and editing (supporting). **Bryan Hughes:** formal analysis (supporting), project administration (supporting), visualization (lead), writing – review and editing (lead). **Jeff Bowman:** conceptualization (lead), funding acquisition (lead), project administration (lead), resources (lead), supervision (lead), writing – review and editing (lead).

## Funding

This work was supported by the Natural Sciences and Engineering Research Council of Canada.

## Conflicts of Interest

The authors declare no conflicts of interest.

## Supporting information


**Figure S1:** The flyer used to advertise the small mammal campaign, calling for observation submissions of the eight focal species.
**Figure S2:** The email script advertising the small mammal campaign, delivered to naturalist groups and environmental organizations.
**Figure S3:** Community science: big insights from small mammal data classification system.
**Figure S4:** Across the eight species of focus in this study, no observable trend changes were seen in total observations (A) on iNaturalist submissions from May—August in 2019–2025. Proportional change is also documented (B) in this figure and shows similar patterns to changes in total observations, with a notable increase in least weasel proportional change in observations in 2023.

**Data S1:** ece373506‐sup‐0002‐DataS1.zip.

## Data Availability

All the required data are uploaded as Supporting Information—S1.
