# Supplementary material for: Community Science: Big Insights From Small Mammal Data
Source: Ecol Evol. 2026 Apr 17;16(4):e73506. doi: 10.1002/ece3.73506 (PMC13090111; doi:10.1002/ece3.73506)
Supplement: Supplementary file 1 — Figure S1: The flyer used to advertise the small mammal campaign, calling for observation submissions of the eight focal species. Figure S2: The email script advertising the small mammal campaign, delivered to naturalist groups and environmental organizations. Figure S3: Community science: big insights from small mammal data classification system. Figure S4: Across the eight species of focus in this study, no observable trend changes were seen in total observations (A) on iNaturalist submissions from May—August in 2019–2025. Proportional change is also documented (B) in this figure and shows similar patterns to changes in total observations, with a notable increase in least weasel proportional change in observations in 2023. [file ECE3-16-e73506-s001.docx]

IN SUPPLEMENT:

Figure S1. The flyer used to advertise the small mammal campaign, calling for observation submissions of the eight focal species.


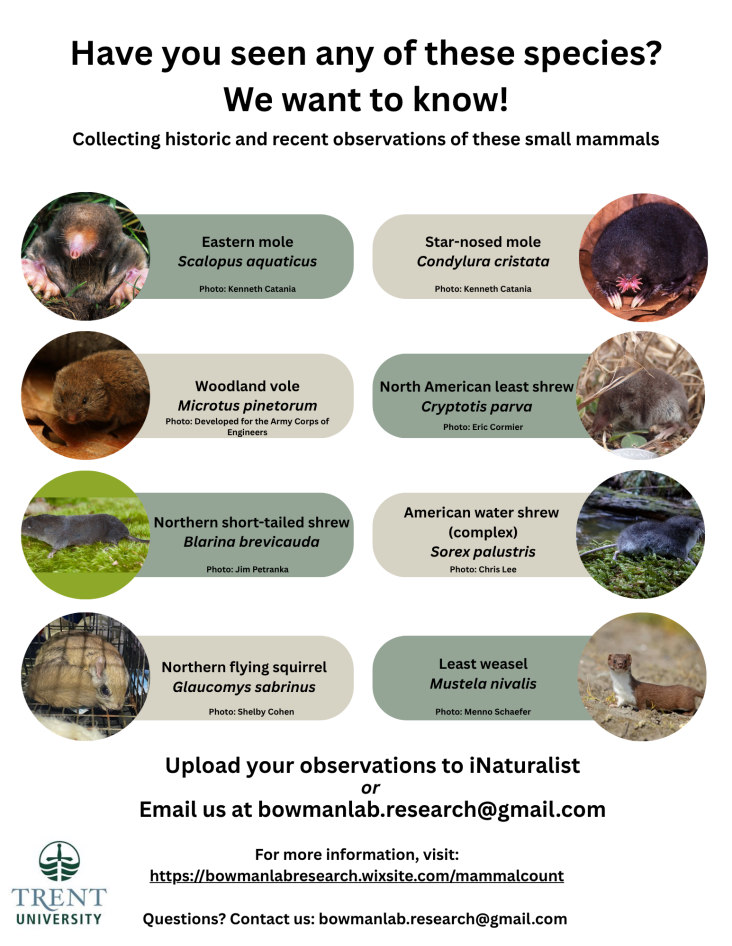


Figure S2. The email script advertising the small mammal campaign, delivered to naturalist groups and environmental organizations.


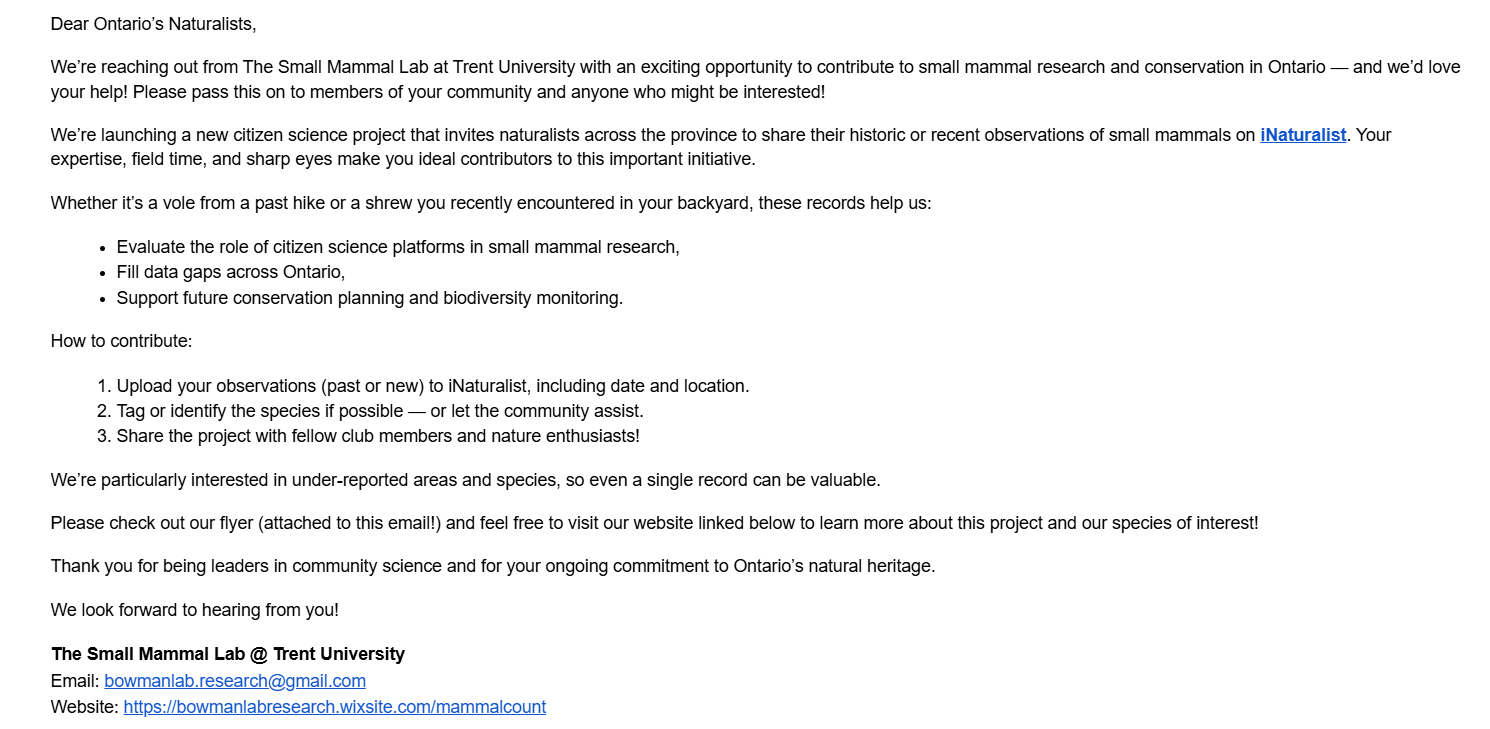


Figure S3. **Community science: big insights from small mammal data classification system.**


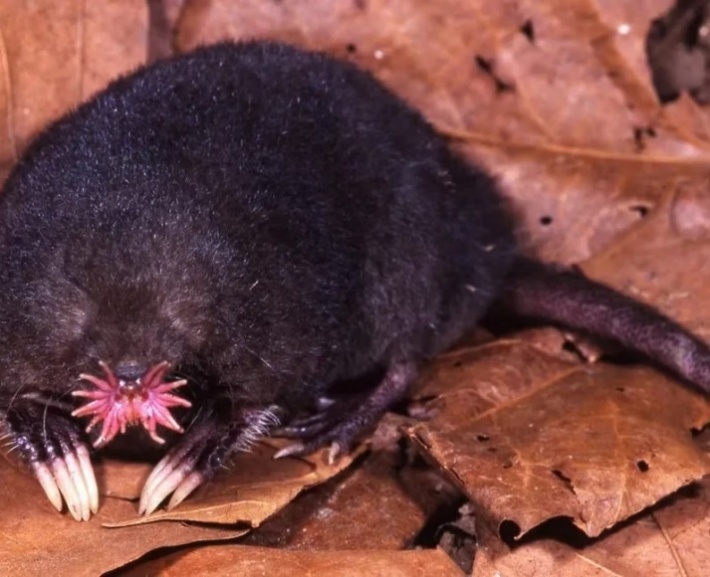


**Star-nosed mole:** *Condylura cristata.* Image by Kenneth Catania.

**Rarity classification:** NHIC classifies the star-nosed mole as S5; thus, we classified this species as abundant.

**Identity classification:** We designated the star-nosed mole as non-confusible since individuals have a unique, identifiable morphological figure. The hairless nose, which contains 22 fleshy tentacles that loosely form the shape of a star.


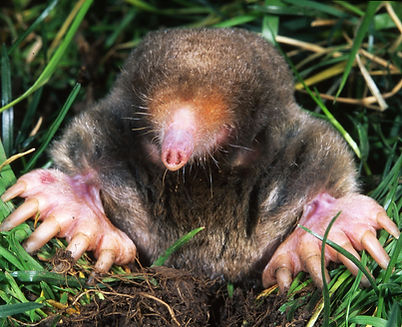


**Eastern mole:** *Scalopus aquaticus.* Image by Kenneth Catania.

**Rarity classification:** NHIC classifies the Eastern mole as S2; thus, we classified this species as rare.

**Identity classification:** We designated the eastern mole as confusible. Although eastern moles are characterized by large feet, a short hairless tail, and ears covered by fur, these features are not easily identifiable from photos. Likewise, other species, such as the hairy-tailed mole (*Parascalops breweri*), overlap in habitat and look similar, making identification difficult without prior experience.


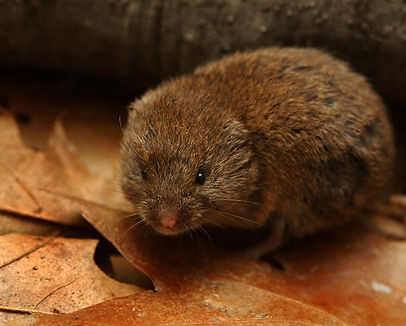


**Woodland vole:** *Microtus pinetorum*. Photo developed from the Army Corps of Engineers.

**Rarity classification:** NHIC classifies the woodland vole as S3; thus, we considered this species rare.

**Identity classification:** The woodland vole is a small woodland mammal with reddish-brown fur. We classified this species as confusable because several other small mammals, including lemmings, mice, and other voles with a similar fur colour, can be commonly mistaken for the woodland vole without prior knowledge.


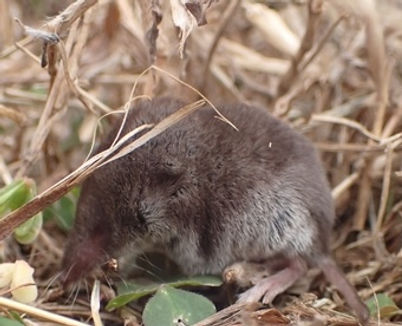


**Least shrew:** *Crptotis parva* photo by Eric Cormier

**Rarity classification:** NHIC classifies the least shrew as SH; thus, we classified the least shrews as rare.

**Identity classification:** The woodland vole is small (7.5 – 9 cm in length), with a short tail, dark brown dorsal fur, and ashy white ventral fur. We classified this species as confusable because many shrews overlap in habitat and share a similar pelage colour scheme. Even trained ecologists often rely on skull and tooth morphology or genetic analyses to correctly identify shrews.


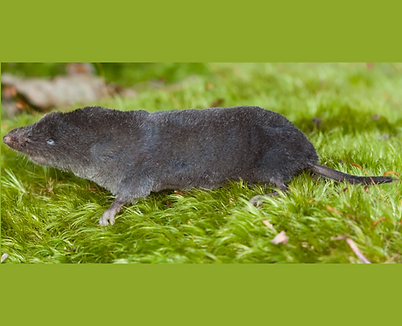


**Northern short-tailed shrew:** *Blarina brevicauda* photo by Jim Petanka.

**Rarity classification:** NHIC classifies the northern short-tailed shew as S5; thus, we classified the northern short-tailed shrew as abundant.

**Identity classification:** We classified the northern short-tailed shrew as non-confusible. Although shrews are often difficult to distinguish, the northern short-tailed shrew is the largest in Ontario (10-13 cm) and has a noticeably short tail and slender body form.


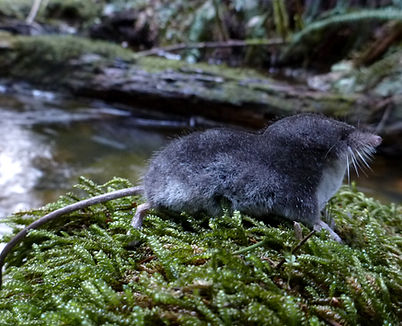


**American water shrew (complex):** *Sorex palustis* photo by Chris Lee

**Rarity classification:** NHIC classifies the water shrews as S5; therefore, we considered these species to be abundant.

**Identity classification:** We classified the American water shrews as confusible. Water shrews are characteristically found in bogs, streams, and ponds. Large, webbed feet, with stiff hairs along the sides and a bicoloured tail with a dark top and lighter underside, are common morphological features used to identify water shrews. However, these characteristics are difficult to see in photography and are unlikely to be common knowledge for anyone without prior experience identifying shrews.


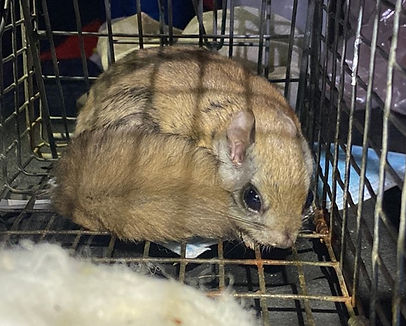


**Northern flying squirrel,** *Glaucomys sabrinus***,** photo by Shelby Cohen.

**Rarity classification:** NHIC classifies the Northern flying squirrels as S5; therefore, we considered this species as abundant.

**Identity classification:** Northern flying squirrels are easily identifiable as flying squirrels (as opposed to other squirrels) by their characteristic gliding behaviour and the presence of a patagium. However, we classified this species as confusable, since northern and southern flying squirrels (Glaucomys volans) overlap in habitat. The two species are often distinguished by size and ventral fur, where northern flying squirrels' ventral fur is grey at the base, compared to the all white ventral fur of the southern flying squirrel. Size and fur colour are difficult to distinguish in photographs; likewise, without prior knowledge of how to identify flying squirrels, untrained individuals would likely have difficulty differentiating these two species.


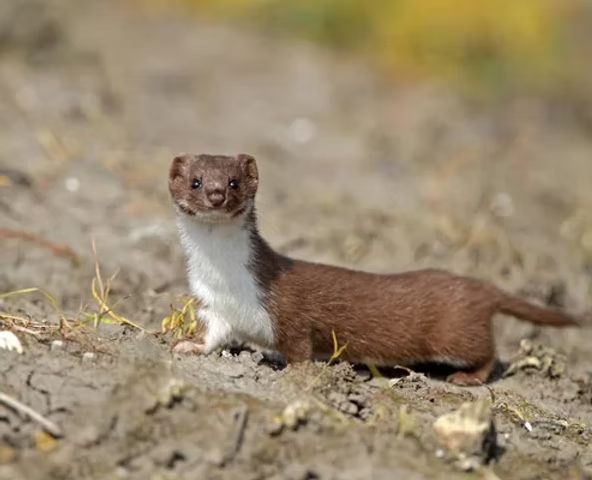


**Least weasel,** *Mustela nivalis***,** photo by Menno Schaefer.

**Rarity classification:** NHIC classifies the least weasel as SU; thus, we classified them as rare.

**Identity classification:** The least weasel is a small mustelid (about 20 cm long) with brown dorsal fur and white ventral fur in the summer, with a pure white coat in the winter. We classified this species as non-confusible, since the characteristic black tip of its tail is easily seen in photos and is unique to this species.


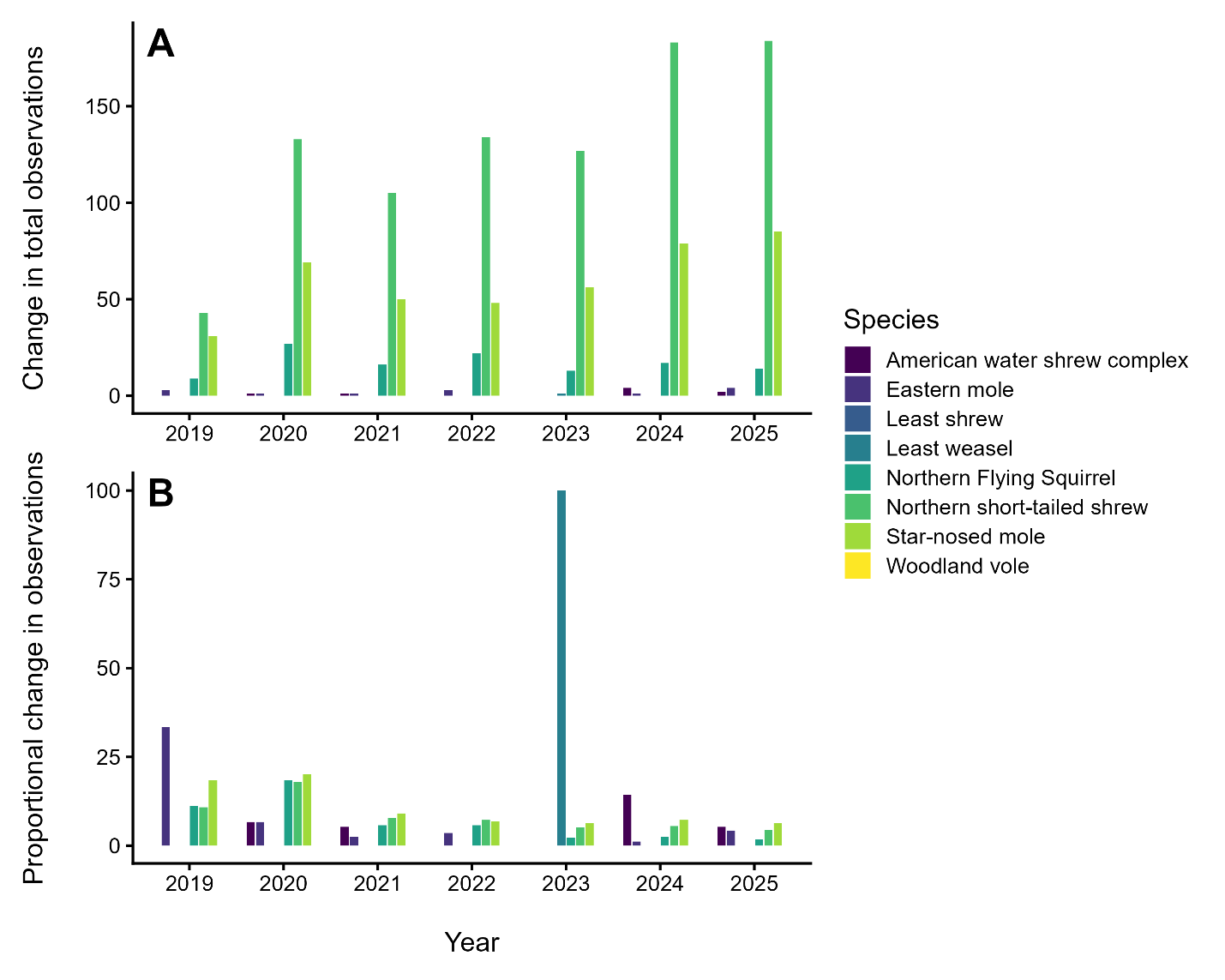


Figure S4: Across the eight species of focus in this study, no observable trend changes were seen in total observations (A) on iNaturalist submissions from May – August in 2019 – 2025. Proportional change is also documented (B) in this figure and shows similar patterns to changes in total observations, with a notable increase in least weasel proportional change in observations in 2023.
